# Supplementary figures and images for: An atypical EhGEF regulates phagocytosis in Entamoeba histolytica through EhRho1
Source: PLoS Pathog. 2021 Nov 22;17(11):e1010030. doi: 10.1371/journal.ppat.1010030 (PMC8648123; doi:10.1371/journal.ppat.1010030)

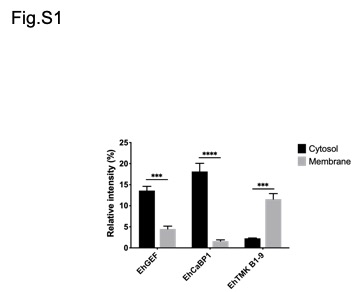

Supplement: S1 Fig — Quantitative analysis of immunoblots of subcellular fractionation from Fig 3A using software AlphaEaseFC 4.0 based on three independent experiments. (TIF) [file ppat.1010030.s001.tif]

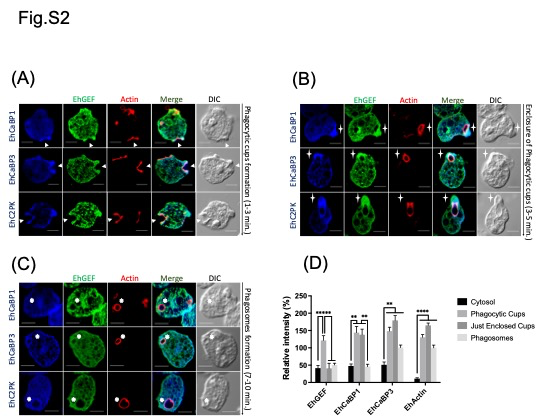

Supplement: S2 Fig — Co-localization was studied in immunostained images of EhGEF during erythrophagocytosis assay with indicated phagocytic marker proteins during (A) Initiation of phagocytic cup formation at (1–3) minutes (B) Progression of phagocytic cups at (5–7) minutes and (C) Phagosome formation at (7–10) minutes. 48h grown E. histolytica cells were incubated with RBC for different time intervals at 37°C and subsequently fixed for immunostaining. Cells were immunostained with HA-tag specific antibody followed by Alexa-488 conjugated secondary antibody (green), F-actin was stained with TRITC conjugated phalloidin (red). EhCaBP1, EhCaBP3 and EhC2PK were immunostained with protein specific antibodies followed by Alexa-405 conjugated secondary antibody (blue). Arrowheads indicate phagocytic cups with enrichment of indicated proteins. (D) Quantitative analysis of fluorescent signals of indicated proteins in different steps of phagocytosis in immunostained images of E. histolytica cells. Four regions were selected from cytosol, phagocytic cups, just closed cups and phagosomes for each cells. Average intensity was calculated for each region. Relative intensities were calculated by assuming intensity as 100% for each marker separately. This experiment was carried out by selecting randomly five cells in triplicates. (N = 5, bar represent standard error). Bar represent 10μm, DIC is differential interference contrast. (F) Co-localization coefficient was analyzed from 10 cells using NIS 4.0 AR software. PCC(r) value of EhGEF with EhCaBP1, EhC2PK and EhCaBP3 during phagocytic cup formation is indicated. ANOVA test was used for statistical comparisons.*p-value≤0.05, **p-value≤0.005, ***p-value≤0.0005. (TIF) [file ppat.1010030.s002.tif]

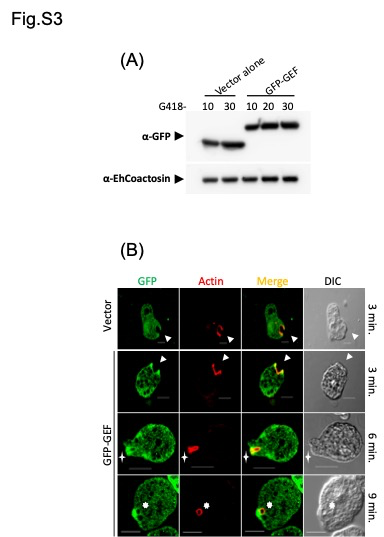

Supplement: S3 Fig — (A) Western analysis of GFP -EhGEF. E. histolytica cell expressing GFP-EhGEF were induced with different concentration of G418(10, 20 and 30 μg/ml) for 48h. Each lane was loaded with 100 μg of cell lysate as shown in figure. Blot probe with anti-GFP antibody and EhCoactosin was taken as a loading control for experiment. (B) Fluorescence images of E. histolytica cells expressing GFP-EhGEF during phagocytosis of RBC (Red). Cell were induced as mention above and immunostained with anti GFP tag specific antibodies followed by Alexa 488 and F-actin was stained with TRITC phalloidin. (TIF) [file ppat.1010030.s003.tif]

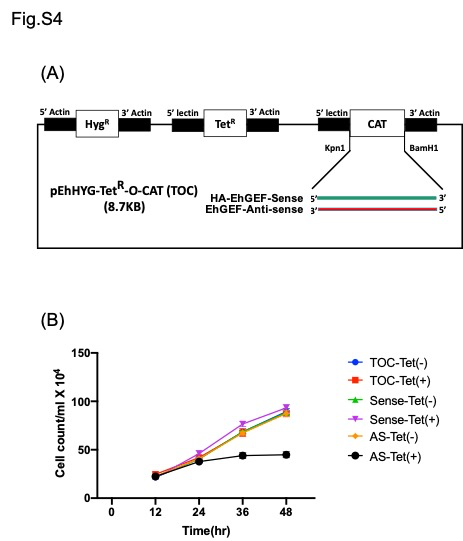

Supplement: S4 Fig — (A) Schematic representation of E. histolytica specific tetracycline inducible pEhHyg-TetR-O-CAT (TOC) vector. EhRho1 was cloned in sense and antisense orientation in BamH1 and Kpn1 sites of pEhHyg-TetR-O-CAT vector. (B) Proliferation of E. histolytica trophozoites carrying different constructs was studied. All cells were grown in presence of 10 μg/ml hygromycin and tetracycline was added to the medium at 30 μg/ml at starting time. Cells were grown in 5 ml culture tubes in triplicate for all the experiments and counting was carried out using a haemocytometer, after chilling the tube for 5 min. One-way ANOVA test was used for statistical comparisons. (TIF) [file ppat.1010030.s004.tif]

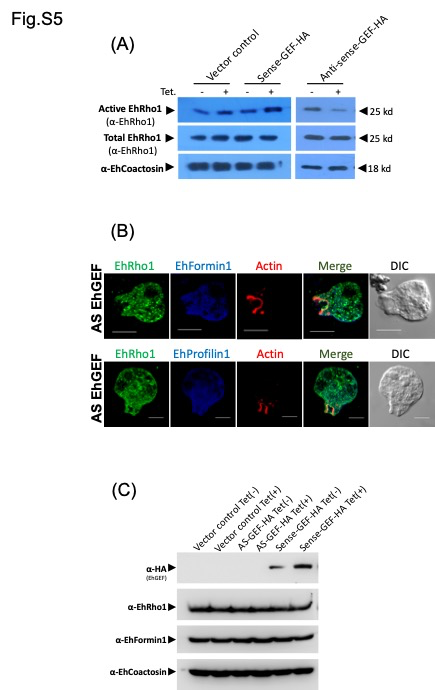

Supplement: S5 Fig — (A) Rhotekin GST-Rho-binding domain (RBD) pull-down assay. Glutathione sepharose beads bound to GST-tagged Rho-binding domain of Rhotekin was incubated with indicated cell lysate for 4 hr during which the activated EhRho1 binds to GST-RBD. The beads were washed three times before analysis by western blotting. For internal and loading control, 100 μg of lysate was taken prior to incubation of beads with the lysate. The activated EhRho1 decreases markedly in EhGEF antisense cells. (B) Immunofluorescence images of E. histolytica cells expressing anti-sense EhGEF during erythrophagocytosis. Cells were stained with anti-EhRho1, anti-EhFormin1 or anti-EhProfilin1 antibodies followed by Alexa-405 or Alexa-488 secondary antibodies. Actin was stain with TRITC-phalloidin. Bar represent 10μm, DIC is differential interference contrast. (C) Western blot analysis of amoebic cells expressing indicated constructs showing the level of EhRho1 in vector alone, antisense (AS) and sense EhGEF in the presence and the absence of tetracycline. EhCoactosin1 was used as an internal and loading control. (TIF) [file ppat.1010030.s005.tif]

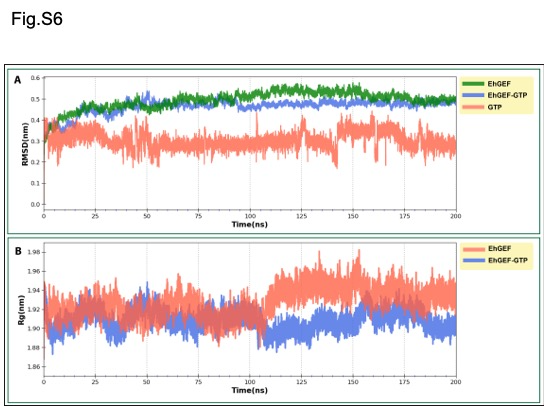

Supplement: S6 Fig — (A) The equilibrated RMSD profile shows no significant conformational changes in the modelled EhGEF and also the EhGEF-GTPcomplex. (B) The radius of gyration showing the compactness of EhGEF and EhGEF-GTPcomplex. (TIF) [file ppat.1010030.s006.tif]

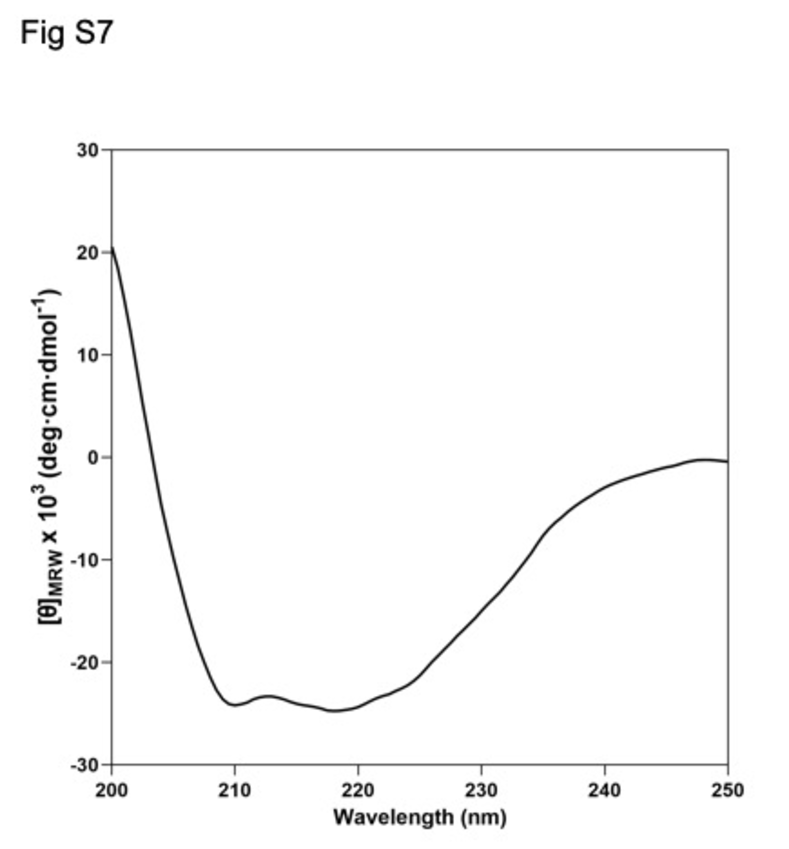

Supplement: S7 Fig — The CD spectra confirms the α/β content of secondary structure as predicted from the sequence information and modelled structure. (TIF) [file ppat.1010030.s007.tif]
