# Supplementary material for: An atypical EhGEF regulates phagocytosis in Entamoeba histolytica through EhRho1
Source: PLoS Pathog. 2021 Nov 22;17(11):e1010030. doi: 10.1371/journal.ppat.1010030 (PMC8648123; doi:10.1371/journal.ppat.1010030)
Supplement: S1 Table — The proteins are arranged in order of sequence coverage in the mass spectrometry data. (DOCX) [file ppat.1010030.s008.docx]

| EhRho1 Binding proteins | | |
| --- | --- | --- |
| Acc. No. | Name | % Coverage |
| *C4LYE4* | *Putative uncharacterized protein OS=Entamoeba histolytica GN=EHI_008090 PE=4 SV=1 - [C4LYE4_ENTHI]* | *14.94* |
| *B1N368* | *Putative uncharacterized protein OS=Entamoeba histolytica GN=EHI_065490 PE=4 SV=1 - [B1N368_ENTHI]* | *12.17* |
| *C4M711* | *Putative uncharacterized protein OS=Entamoeba histolytica GN=EHI_189930 PE=4 SV=1 - [C4M711_ENTHI]* | *12.16* |
| C4LY96 | Putative uncharacterized protein OS=Entamoeba histolytica GN=EHI_166920 PE=4 SV=1 - [C4LY96_ENTHI] | 11.99 |
| *O15608* | *Ras-related protein RAB7 (Fragment) OS=Entamoeba histolytica PE=2 SV=1 - [O15608_ENTHI]* | *11.83* |
| Q24817 | Rho-related protein racD (Fragment) OS=Entamoeba histolytica GN=RACD PE=2 SV=1 - [RACD_ENTHI] | 8.59 |
| *C4M344* | *C2 domain containing protein OS=Entamoeba histolytica GN=EHI_059860 PE=4 SV=1 - [C4M344_ENTHI]* | *8.65* |
| *B1N2Q5* | *Actin-related protein 2/3 complex subunit 5 OS=Entamoeba histolytica GN=EHI_103440 PE=3 SV=1 - [B1N2Q5_ENTHI]* | *9.56* |
| *B1N309* | *Rab GDP dissociation inhibitor alpha, putative OS=Entamoeba histolytica GN=EHI_167060 PE=4 SV=1 - [B1N309_ENTHI]* | *7.06* |
| *B1N354* | *Ras-1, putative OS=Entamoeba histolytica GN=EHI_124520 PE=4 SV=1 - [B1N354_ENTHI]* | *7.00* |
| *O76754* | *Rho GDP exchange inhibitor (Fragment) OS=Entamoeba histolytica GN=Rhoxi PE=2 SV=1 - [O76754_ENTHI]* | *6.55* |
| *C4LY07* | *Ras GTPase activating protein, putative OS=Entamoeba histolytica GN=EHI_035800 PE=4 SV=1 - [C4LY07_ENTHI]* | *5.21* |
| *C4M3P4* | *Myosin heavy chain OS=Entamoeba histolytica GN=EHI_140720 PE=4 SV=1 - [C4M3P4_ENTHI]* | *3.28* |
|  |  |  |

S1_Table

| EhGEF Binding proteins | | |
| --- | --- | --- |
| Acc. No. | Name | % Coverage |
| *Q8I893* | Rho-like small GTPase OS=Entamoeba histolytica GN=Rho PE=2 SV=1 - [Q8I893_ENTHI] | 83.49 |
| *Q9TYD6* | Actin (Fragment) OS=Entamoeba histolytica PE=2 SV=1 - [Q9TYD6_ENTHI] | 70.78 |
| *B1N2Q5* | Actin-related protein 2/3 complex subunit 5 OS=Entamoeba histolytica GN=EHI_103440 PE=3 SV=1 - [B1N2Q5_ENTHI] | 58.09 |
| C4M4P4 | Actin-binding protein, cofilin/tropomyosin family OS=Entamoeba histolytica GN=EHI_168340 PE=4 SV=1 - [C4M4P4_ENTHI] | 51.35 |
| C4LU72 | Myosin heavy chain OS=Entamoeba histolytica GN=EHI_110180 PE=4 SV=1 - [C4LU72_ENTHI] | 51.14 |
| C4LSV0 | ARP2/3 complex 20 kDa subunit, putative OS=Entamoeba histolytica GN=EHI_030820 PE=4 SV=1 - [C4LSV0_ENTHI] | 47.12 |
| Q76N78 | Profilin (Fragment) OS=Entamoeba histolytica PE=2 SV=1 - [Q76N78_ENTHI] | 40.91 |
| O76754 | Rho GDP exchange inhibitor (Fragment) OS=Entamoeba histolytica GN=Rhoxi PE=2 SV=1 - [O76754_ENTHI] | 33.93 |
| Q9BLF3 | Rab family GTPase OS=Entamoeba histolytica GN=EhRab1A PE=2 SV=1 - [Q9BLF3_ENTHI] | 31.71 |
| C4LWM0 | C2 domain containing protein OS=Entamoeba histolytica GN=EHI_069320 PE=4 SV=1 - [C4LWM0_ENTHI] | 31.38 |
| O76321 | Rho-related protein racG OS=Entamoeba histolytica GN=RACG PE=3 SV=1 - [RECG_ENTHI] | 16.08 |
| C4LY07 | Ras GTPase activating protein, putative OS=Entamoeba histolytica GN=EHI_035800 PE=4 SV=1 - [C4LY07_ENTHI] | 7.75 |
